# Effect of maternal dietary habits and gestational weight gain on birth weight: an analytical cross-sectional study among pregnant women in the Tamale Metropolis

**DOI:** 10.11604/pamj.2023.44.19.38036

**Published:** 2023-01-11

**Authors:** Abdulai Abubakari, Mubarick Nungbaso Asumah, Nihad Zimpa Abdulai

**Affiliations:** 1 Department of Global and International Health, School of Public Health, University for Development Studies, Tamale Northern Region, Tamale, Ghana,; 2 Ghana Health Service, Kintampo Municipal Hospital, Kintampo Bono East, Bono, Ghana,; 3 Department of Nutritional Sciences, School of Public Health, University for Development Studies, Tamale Northern Region, Tamale, Ghana

**Keywords:** Birth weight, determinants, dietary patterns, gestational, weight gain, maternal dietary habits, postnatal mothers

## Abstract

**Introduction:**

dietary intake and optimal gestational weight gain are important factors leading to a positive outcome for both mothers and their infants. Women who consume inadequate diet and gain inadequate weight during pregnancy are at risk of bearing a baby with low birth weight, whereas those who gain excessive weight are at increased risk of preeclampsia, having macrosomal babies, and gestational diabetes. The study aimed to assess the effect of maternal dietary intake, gestational weight on birth weight among pregnant women in Tamale Metropolis.

**Methods:**

the study was a health-facility-based analytical cross-sectional study that involved 316 postnatal mothers. A semi-structured questionnaire was used to collect the data. Data collected were analyzed using STATA version 12. Multiple logistic regression model was estimated to determine the predictors of birth weight. Statistical significance was set at p<0.05.

**Results:**

the study showed 17.8%, 55.9%, and 26.4% prevalence of inadequate, adequate, and excessive gestational weight gain, respectively. Although, all respondents consume supper every day, only 40.0% consumes snacks daily, 97.5% and 98.7% consumes breakfast and lunch daily respectively. Majority of the respondents (92.4%) had acceptable minimum dietary diversity. About 11.0% and 4.0% of the babies were low birth weight and macrosomic, respectively. Furthermore, the prevalence of inadequate and adequate dietary intake was, respectively, 7.6% and 92.4%. The results showed that underweight before pregnancy (BMI<18Kg/m^2^) (AOR=8.3, 95% CI: 6.7-15.0) and inadequate weight gain during pregnancy (AOR=4.5, 95% CI: 3.9-6.5) were significant determinant of low birthweight baby.

**Conclusion:**

on the whole, maternal body mass index and weight gain during pregnancy were strong predictors of low birth weight. Low birth weight is a major public health concern and the causes multifaceted in natures. Therefore, to deal with low birth weight, a more holistic and multi-sectoral approaches such as behaviour change communication and comprehensive preconception care are required.

## Introduction

Malnutrition in pregnancy, especially in developing countries, is at the centre of many women's health issues [1]. During pregnancy, dietary intake is of great significance as far as fetal growth and development are concerned [2]. Consuming a variety of food groups by pregnant women reduces their risk of giving birth to low birth weight babies [3]. Poor nutrition in pregnancy is related to poor birth outcomes including low birth weight babies, preterm delivery, and intrauterine growth retardation [4]. In the same way, a healthy birth outcome stems from a good nutritional status [5] therefore the diet should be diverse and balanced for pregnant women [6].

In developing nations, malnutrition in pregnant women is often the result of insufficient dietary intake, poverty, poor healthcare system, heavy workload, recurrent infections, and lack of knowledge about the appropriate food to take during pregnancy [7]. Dietary intake in pregnancy is complicated by physiological changes which lead to poor dietary intake, which has negative consequences on the developing fetus and the mother [8]. For example, iron deficiency anemia in pregnant mothers is closely linked to low birth weight in newly born infants coupled with a greater risk of premature birth [9]. Moreover, malnutrition is related with retarded growth and protein-energy malnutrition due to insufficient dietary intake, especially throughout childbirth [10].

Gaining a healthy weight appropriate for pregnancy historically is a problem for women due to insufficient nutrition [11]. However, over the past few decades, excessive weight gain has become prevalent globally, affecting all age groups, including pregnant women [12]. Obesity, a worldwide phenomenon is considered more sensitive in women than in men. This is because there are heightened dietary needs during pregnancy that are important for the mother and the baby's health [13]. A population's health status can be assessed using the number of babies born with low birth weight (<2500g) [14]. Stunting and non-communicable diseases as well as child survival are link to low birthweight, therefore, there is a need for effective public health interventions that address low weight at birth [15,16]. According to United Nations Children's Fund (UNICEF) [17], the prevalence of low birth weight (LBW) in Ghana is 14.2%. The prevalence, however, is higher in Northern Ghana than the southern part of Ghana. For example a study by Abubakari *et al*. [18] in Northern Ghana reported low birth weight as 26% whiles Fosu *et al*. [19] found a prevalence of 21%, in the Brong Ahafo. Being “too big” at birth (macrosomia) is also considered an undesirable pregnancy outcome with associated elevated risk to both mother and child [20]. However, in developing countries, macrosomia is given less consideration [21].

Birthweight more than or equal to 4.0kg is regarded as macrosomic birth [22,23]. The prevalence of macrosomia is between 5% and 20% in developed countries [24]. However, the prevalence of macrosomia in developing countries appears to be sporadic. For instance, in Lu *et al*. [22] study, a rise from 6.0 percent in 1994 to 7.8 percent in 2005 was recorded. A recent study on macrosomic births amongst women who were obese and overweight reported a 10.9% prevalence rate [25]. Macrosomia, which is associated with obesity and far occurs ahead of adult life [26], may lead to complex birth outcomes [23]. Obesity could lead to additional risks to mothers and newborns in resource-limited countries such as Ghana.

During pregnancy, weight gain has major health implications for both mother and infant [27]. Inadequate weight gain is related to the mother´s and fetus´ health risks [28]. Insufficient gestational weight gain could lead to low birth weight and preterm childbirth [29]. Adverse maternal and fetal effects such as large for gestational age (LGA) babies, gestational diabetes mellitus (GDM), caesarean delivery, early pregnancy loss, preeclampsia, and postpartum weight retention result from excessive weight gain during pregnancy [30]. Optimal gestational weight gain contributes to positive pregnancy outcomes such as optimal growth and development of the fetus and reduced probability of mortality during pregnancy [31]. The prevalence of inadequate and excessive gestational weight gain, birth outcome and associated risk factors vary from one setting to another.

One of the most significant causes of maternal and child morbidity and mortality, especially in developed countries, is maternal malnutrition [32]. Undernutrition among pregnant women in Ghana decreased from 9 percent in 2003 to 6 percent in 2014, whereas overnutrition increased from 26 percent in 2003 to 40 percent in 2014 [33]. Among the factors that influence the nutritional status of the developing fetus, maternal nutrition is an important one [34], because a vital inter-dependence can be found amongst the overall nutritional health of the expectant mother and that of the fetus health [35]. Therefore, the nutrition, and health of women are of decisive importance during the entire period of pregnancy.

The prevalence of excessive gestational weight gain is rising and alarming, indicating a public health concern [12]. Numerous studies in developed countries on gestational weight gain indicated that more than 40% of women are gaining weight above the Optimal Institute of Medicine (IOM) ranges [12]. It is further estimated that over 75% of African American women of reproductive age are overweight or obese and this increases their already high risk for obesity-related adverse pregnancy outcomes [36].

Optimal nutrition during pregnancy is essential for the health of the mother and the developing fetus. Dietary intake is usually predicted by culture and by the type of food available for consumption in a particular locality; hence findings in one locality may not apply to all localities in Ghana. As far as this study is concerned, a review of literature showed that there are limited studies investigating the dietary habits of pregnant women in the Tamale Metropolis and their resulting relationship with birth weight. Therefore, this study aims at investigating the maternal dietary habits, gestational weight gain and their effects on birth weight in the Tamale Metropolis. Specifically, the study will assess the dietary pattern of pregnant women, prevalence of gestational weight gain, prevalence of birth weight and associated risk factors in the Tamale Metropolis.

## Methods

**Study area:** the research was conducted in the Tamale Metropolis. The metropolis has a gross approximate proportion of land of 646.90180 sqkm [37]. Geologically, near latitudes 9^0^16^1^ and 9^0^34^1^ North and longitudes 0^0^36^1^ and 0^0^57^1^ West of the metropolitan area. The inhabitants of the metropolis are made up of 223,252. The percentage of females (50.2%) is higher compared to that of males (49.7%) [37].

**Study design:** analytical cross-sectional study design was used. The design aided in collecting data at a point and identification of relations concerning the different variables.

**Study variables:** the study considered birth weight and dietary habits as dependent variables and all other factors as independent.

**Study population:** the study population was postnatal mothers who are permanently residing in the Tamale Metropolis of the northern region.

**Inclusion criteria:** postnatal mothers who were willing to participate in the study and are permanent residents of the Tamale Metropolis and have sound minds and health were included in the study.

**Exclusion criteria:** excluded from the study were postnatal mothers with chronic conditions such as cancer and diabetes. This is because these factors are believed to affect an individual's nutritional status. The respondent's clinical reports in the maternal and child health booklet and medical documents were used to collect this health information.

**Sample size determination:** the sample size was determined based on a Snedecor and Cochran formula [38]:


$$n=\frac{Z^{2}*p(1-p)}{m^{2}}$$

Where n=sample size, Z is the z-score of a 95% confidence level equivalent to 1.96, and p=prevalence of gestational overweight was 25.1% [18]. Where n is the sample size, Z (statistic) = 1.96, p (prevalence) = 0.29, d (margin of error) = 0.05.


$$n=\frac{{\left(1.96\right)}^{2}*0.251(1-0.251)}{0.05^{2}}=288$$

Using a 10% non-compliance and response rate, the sample size was estimated as 316. Thus, the total number of mothers to be recruited in this study is 316.

**Sampling method:** the study used a multistage sampling approach. The first stage used random sampling to select the health facilities in the metropolis. Consecutive sampling was used to select the study participants. On a typical data collection day, all women in attendance at the various postnatal points of the selected hospitals were approached and eligible candidates were made to sign the consent form and interviewed. This procedure was repeated on each day of data collection until the required sample size was obtained.

**Data collection and instruments:** the data collection was done within two months (March 2020 to May 2020). Semi structured questionnaire was used to interview respondents. The interviews were conducted in the local language (Dagbanli) for respondents who could not read and write in English or chooses to communicate in the local language. The semi-structured questionnaire was divided into sections: section A covered socio-demographic characteristics; section B covered household assets and wealth; section C covered was a food frequency questionnaire adapted from the Ghana Demographic and Health Survey report and modified to suit the purpose of the research. A check list was also developed to capture obstetric history including weigh measurements from first antenatal care (ANC) visit up to delivery and birth weight.

The socio-demographic information generated included: age, parity, marital status, educational level, and core livelihood of the respondent and the husband of married/socio-segment data essentials were: age, equality, marital status, literacy level, and center business of the respondent and the spouse whenever wedded. In assessing pregnant women's nutritional status, methods such as the use of anthropometrics was used [39]. In developing countries, the use of body mass index (BMI) in assessing nutritional status among pregnant women has been restrained since most women show up at their prenatal hospital late and so their pre-pregnancy BMI might stay unidentified [40].

### Definition of variables

**Ideal weight gain:** the expansion of organs in lactation, growth of the fetus, and body fluid increment in volume is a result of the unceasing weight gain in pregnancy. During pregnancy, an increment of 25.0% initial body weight is considered normal. Therefore, for an average pregnant woman to reach this 25.0% initial body weight, she should gain weight of 10kg during pregnancy. An ideal weight gain is 25.0% of the initial body weight (approximately 10kg) and a lower body weight gain could be as a result of greater loss of body fat due to inadequate intake of food or a smaller gain in cell mass and extracellular fluid.

**Guidelines for determining gestational weight gain:** the guideline for weight gain in pregnancy was proposed by the Institute of Medicine (IOM) in the year 1990. This enabled the promotion of adequate weight gain in gestation to avert premature births. While objectives were not being entirely achieved, the Institute of Medicine guidelines for pregnancy weight gain was reorganized in 2009 to use standard body mass index (BMI) groupings which were developed by the World Health Organization (WHO) with a shift towards better maternal health outcomes [41]. The guidelines set out had three categories of gestational weight gain specifically; low or inadequate, normal or adequate, and excessive gestational weight gain. These classifications are based on the pre-pregnancy BMI.

In all antenatal care visits, the weights of expectant mothers are recorded and used as a significant clinical test in antenatal care to monitor fetal growth and development. Weight gain of mothers is also a measurement often discussed by the pregnant woman in session with her doctor or midwife. When the pregnant woman does not gain weight as the pregnancy progresses, there is a need to raise a concern. The WHO developed and instituted the recommended weight gain during pregnancy, which was based on pre-pregnancy weight. Weight gained during pregnancy was determined by subtracting the weight at first ANC from the weight at last ANC and categorized according to the IOM recommendation.

**Measurement of birth weight and pre-pregnancy BMI:** according to the World Health Organization, a low birth weight infant is one born with a birth weight of <2.5kg, while macrosomia is defined as birth weight ≥4.0kg [42]. Therefore, this study considered 3 categories of birth weight established by the World Health Organization which are: (1) a birth weight <2.5kg indicating low birth weight babies; (2) normal birth weight (≥2.5kg but less than 4.0 kg) and; (3) macrosomia (≥4.0kg) [42]. Pre-pregnancy BMI was determined using the weight measured during the first trimester as an appropriate proxy of pre-pregnancy weight [25,43] as weight gain during the first trimester is low (averagely 1 kg) [43]. The BMI was classified into four: (1) underweight (BMI <18.5kg/m^2^); (2) normal weight (18.5kg/m^2^to 24.9kg/m^2^); (3) overweight (25kg/m^2^to 29.9kg/m^2^) and; (4) obese (≥30kg/m^2^).

**Measurement of individual dietary diversity score:** the study aimed to assess the nutrient adequacy of the usual diet consumed during pregnancy. Respondents reported the frequency of consumption of each food on a daily, weekly and monthly basis and received 1 point if they consumed any food within the food group at least once a week during the period of pregnancy and 0 points if they rarely consumed (once a month)/never consumed the food (both in and out of home). The frequency of consumption of various food groups was analyzed by adopting the guidelines of the Food and Agricultural Organization of the United Nations for measuring household or individual dietary diversity [44]. Minimum dietary diversity for women (MDD-W) was used. Foods eaten by the respondents were classified into 10 food groups: starchy staples (cereals and white roots and tubers); dark green leafy vegetables; other vitamin A-rich fruits and vegetables; oils/fats; legumes, nuts, and seeds; other fruits and vegetables; organ meat; milk and milk products; eggs; meat and fish, seafood. The individual dietary diversity score was calculated as the sum of food groups consumed. The total individual food scores were first categorized into three, namely; low MDD-W (1-3 food groups); medium MDD-W (4-5 food groups), and high MDD-W (6 or more food groups). For further analysis, these groups were then dichotomized into two categories where 0-4 were considered low dietary diversity scores and greater than or equal to 5 food groups were considered high dietary diversity scores. The final frequency of consumption of each food group was determined on a daily and occasional basis from all food groups consumed by the women during pregnancy.

**Socio-economic status:** the socio-economic status was determined by summing up all household assets, totaling 14 household assets used in the questionnaire. Then, based on the number of household assets owned, an individual was classified as either low or high depending on the number of assets owned, those who owned less than 7 assets were classified as having low socioeconomic status, and those having 7 or more assets were classified as having high socioeconomic status.

**Data/statistical analysis:** data entry was done using Statistical Package for the Social Sciences (SPSS version 21) and transferred to STATA 12.1 for further analysis. Uniformity and plausibility checks were done after the data entry to ensure that errors were reduced. Descriptive statistics including means and standard deviations (SD) were generated for continuous variables while frequencies and percentages were generated for categorical variables. Independent t-tests were used to analyze the relationships between categorical factors and birth weight means. In cases where there were more than two groups generated, one-way ANOVA was employed to compare means. Explanatory variables under investigation were maternal age, education, occupation, parity, religion, ethnicity, husband's occupation and education, body mass index, and weight gain. The outcome variables were newborn birth weight (low birth weight, normal and macrosomia). Bivariate analyses were done using chi-square test to determine the associations between categorical variables with a statistical significance set at p<0.05. Multiple logistic regression model was estimated to determine the predictors of birth weight. All explanatory variables that were significantly associated with the outcome variable in the bivariate analyses (*P*<0.05) were entered into a multiple logistic regression model. *P*value less than 0.05 was considered statistically.

**Data quality control:** the first step in ensuring data quality was to provide enough training for data collectors. During the training data collectors were taught on how to administer surveys and retrieve secondary data from maternal health records books. They were also taken through how to administer a questionnaire using the appropriate interviewing skills. Because data collecting locations were widely used across the region, the completed questionnaires were collected at the end of each week for safe keeping. This guaranteed that the data obtained was verified for any necessary revisions before the next week. This also assured that the information gathered from the responders remained private from third parties. Before the data was input for analysis, all questionnaires were checked for completeness. The questionnaires were written in the English language and were translated orally into the most common locally spoken languages (mainly Dagbanli). When the questionnaire was translated, precautions were taken to ensure that the keywords or important concepts in the questionnaire were not lost. The questionnaire was originally pretested among ten women at a hospital which was not selected for the study. This was done to identify persons who would fit the requirements before the primary research study began. This was done to verify that the questionnaire conveyed the correct information and elicited the appropriate replies for the research. Before the final administration of the questionnaire, the required errors detected during the pretesting were rectified.

**Ethical clearance and consent to participate:** ethical clearance was obtained from the Research Committee for Human Publications and Ethics (CHRPE), Kwame Nkrumah University of Science and Technology, School of Medical Sciences, and Komfo Anokye Hospital Kumasi (CHRPE/AP/105/20) to guarantee all protocols used in the research procedure. After receiving complete information about the study, participants gave signed informed consent. However, for participants under 16 years, consent was obtained from parents or guardians. Finally, this study was conducted in accordance with the principles of the Declarations of Helsinki.

## Results

**Socio-demographic characteristics:** a total sample of 316 women was used in all analyses. The majority (93.7%) of the respondents reside in urban. The mean age of the respondents was 29.0 ± 5.3. The majority of respondents (90.5%) were married. Only 16.1% of the respondents had no formal education, the remaining 83.86% had some form of formal education. The most dominant religion of the respondents was Islam (75.3%). Slightly above half (52.5%) of the respondents had normal BMI. About 91% of the respondents were living in high socioeconomic status households (Table 1).

**Table 1:** sociodemographic characteristics of respondents

Variables	Categories	Frequency(N)	Percentage (%)
Residence	Rural	20	6.33
	Urban	296	93.67
	<20	26	8.23
	20-30	156	49.37
Age	30-40	125	39.56
	>40	9	2.85
	Married	286	90.51
Marital status	Cohabiting	12	3.80
	Divorce/separated	6	1.90
	Widowed	12	3.80
Education	No formal education	51	16.14
	Primary	41	12.97
	JHS	66	20.89
	SHS/vocational	98	31.01
	Tertiary	60	18.99
Parity	No child	38	12.03
	1-3 children	233	73.73
	4-6 children	45	14.24
Religion	Christian	66	20.89
	Muslim	238	75.32
	Traditionalist	12	3.80
Husbands' education	Primary	8	2.53
	JHS/middle school	18	5.70
	SHS	117	37.03
	Tertiary	155	49.05
	None	18	5.70
Husbands' occupation	Trader	50	15.80
	Employed	210	66.50
	Unemployed	7	2.20
	Farmer	37	11.70
	Others	12	3.80
BMI	Underweight	36	11.39
	Normal	166	52.53
	Overweight	91	28.80
	Obese	23	7.28
Socio-economic status	Low	30	9.49
	High	286	90.51

BMI: body mass index; JHS: junior high school; SHS: senior high school

**Meal pattern and food frequency of respondents:** from the analysis done, 97.5% of the respondents consumed breakfast daily. Almost all respondents 98.7% consumed lunch daily while 40.0% were not consuming snacks daily. However, all respondents indicated taking supper every day. Analysis done on the frequency of food consumption indicated food was more frequently consumed in the second and third trimester compared to the first trimester (Table 2).

**Table 2:** meal pattern and food frequency of respondents

Variable	Variable category	Frequency	Percentage
Meal pattern	Breakfast	308	97.46
	Lunch	312	98.74
	Snack	218	60.00
	Supper	316	100.00
**Frequency of meals consumed/day**			
First trim	Once	40	12.66
	Twice	148	48.84
	Trice	118	37.34
	More than trice	10	3.16
Second trim	Once	7	2.22
	Twice	35	11.08
	Trice	164	51.90
	More than trice	110	34.81
Third trim	Once	1	0.31
	Twice	6	1.90
	Trice	94	29.75
	More than trice	215	65.04

**Dietary patterns of pregnant women:**from the study, it was revealed that the more than half y (58.2%) of the women consumed starchy roots and tubers daily. Almost (97.5%) of all women consumed dark green leafy vegetables daily. Most (81.6%) of the women consumed meat and meat products daily. More than half (65.5%) of the women consumed milk and milk products occasionally, but 34.5% of the study participants consumed milk and milk products daily. Women who consumed cereal daily were more (59.8%) than those who consumed it occasionally (40.2%). Overall, majority of the respondents (92.4%) had acceptable minimum dietary diversity (Table 3).

**Table 3:** dietary patterns of pregnant women

Food group category	Frequency of consumption	
	Daily n (%)	Occasionally n (%)
Starchy staples	184 (58.23%)	132 (41.77%)
Dark green leafy vegetables	308 (97.47%)	8 (2.53%)
Organ meat	258 (81.65%)	58 (18.35%)
Milk and milk products	109 (34.49%)	207 (65.51%)
Cereals	189 (59.81%)	127 (40.19%)
Legumes and pulses	271 (85.76%)	45 (14.24%)
Fruits other vitamin A fruits and vegetables	99 (31.33%)	217 (68.67%)
Grains	275 (87.03%)	41 (12.97%)
Eggs	278 (87.97%)	38 (12.03%)
**Dietary diversity**		
Acceptable	292(92.41%)	
Not acceptable	24 (7.59%)	

**Association between dietary diversity and maternal factors:** in univariate analysis, an association between respondents' age and dietary diversity was established (p=0.010). Respondents who were widowed (100%) had adequate diet diversity while those who were cohabiting (58.3%) had inadequate dietary diversity (p=0.001). Moreover, the educational level of respondents was seen to be associated with dietary diversity (p=0.009). Furthermore, religion of the respondents was associated with dietary diversity of the respondents as 95.5%, 92.4%, and 75.0% of respondents who were Christians, Muslims, and traditionalists, respectively had diversified diets (p=0.048). Lastly, it was observed that husbands' education and occupation had an impact on dietary diversity as these were, respectively, statistically significantly associated with dietary diversity (p=0.015 and 0.001) (Table 4).

**Table 4:** association between dietary diversity and maternal factors

Variable	Category	Adequate n (%)	Inadequate n (%)	p-value
Age	<20	20(77.0%)	6(23.0)	0.010
	20-30	144(92.3)	12(7.7%)	
	30-40	120(96.8%)	4(3.2%)	
	>40	8(88.9%)	1(11.1%)	
Marital status	Married	270(94.4%)	16(5.6%)	0.001
	Cohabiting	5(41.7%)	7(58.3%)	
	Divorce/separated	5(83.3%)	1(16.7%)	
	Widowed	12(100%)	0	
Education	No formal education	41(93.2%)	3(6.8%)	0.009
	Primary	48(100%)	0	
	JHS	55(83.3%)	11(16.7%)	
	SHS/vocational	94(96%)	4(4%)	
	University/polytechnic	54(90%)	6(10%)	
Religion	Christian	63(95.5%)	3(4.5%)	0.048
	Muslim	220(92.4%)	18(7.6%)	
	Traditionalist	9(75%)	3(25%)	
Fathers' occupation	Trader	47(94%)	3(6%)	0.015
	Employed	196(93.3%)	14(6.7%)	
	Unemployed	7(100%)	0(0.0%)	
	Farmer	34(91.9%)	3(8.1%)	
	Others	8(66.7%)	4(33.3%)	
Fathers' education	Primary	8(100%)	0(0.0%)	0.001
	JHS/middle school	11(61.1%)	7(38.9%)	
	SHS	107(91.5%)	10(8.5%)	
	Tertiary	150(96.8%)	5(3.2%)	
	None	16(88.9%)	2(11.1%)	
Socioeconomic status	Low	24(80%)	6 (20%)	0.007
	High	268 (93.7%)	18 (6.3%)	

Chi-square analysis; JHS: junior high school; SHS: senior high school

**Gestational weight gain:** from the study, it was revealed that slightly above half of the respondents (55.9%) gained adequate weight while (26.3%) gained excessive weight and (17.8%) gained inadequate weight.

**Prevalence of low birth weight:** among the respondents, the majority (85.0%) had normal birthweight at delivery while 11.0% and 4.0% respectively had low birth weight and macrosomia.

**Maternal characteristics and birth weight:** among the respondents, the mean birth weight was (3.1 ± 0.1kg). Babies born to mothers who were living in urban areas were on the average slightly heavier (3.1 ± 1.7kg) than those living in rural areas (2.9 ± 0.4kg). Babies of mothers within the age category of 20-30 years were averagely slightly heavier (3.1 ± 2.3kg) compared to those within the age group of 30-40 (3.1 ± 0.4kg), >40 (2.7 ± 0.1kg), and <20 (2.6 ± 0.28kg). Babies of mothers who were married were heavier (3.1 ± 1.7kg) than those who were divorced (2.9 ± 0.16kg), and cohabiting (2.5 ± 0.3kg). Newborns of mothers who had 1-3 children previously were averagely heavier (3.1 ± 1.9kg) compared to babies of mothers who were first-timers (2.8 ± 0.5kg) and those of mothers with 4-6 children (2.9 ± 0.2kg). Babies of mothers who gained optimal weight during the period of pregnancy were averagely slightly heavier (3.2 ± 2.1kg) compared to those of mothers who gained excessive weight (3.1 ± 0.3kg) and inadequate weight (2.6 ± 0.4kg) (Table 5).

**Table 5:** maternal characteristics/factors and birth weight

Variable	Category	Frequency (%)	Means± standard deviation	p-value
Residence	Rural	20(6.33%)	3.1±1.7	0.001^a^
	Urban	296(93.67%)	2.9±0.4	
Age/years	<20	26(8.23%)	2.6±0.28	0.017^b^
	20-30	156(49.37%)	3.1±2.3	
	30-40	125(39.56%)	3.1±0.4	
	>40	9(2.85%)	2.7±0.1	
Marital status	Married	286 (90.51%)	3.1±1.7	0.001^b^
	Cohabiting	12 (3.80%	2.5±0.3	
	Divorce/separated	6 (1.90%)	2.9±0.16	
	Widowed	12 (3.80%)	3.1±0.4	
Education	No education	51 (16.14%)	2.9±0.4	0.020^b^
	Primary	41 (12.97%)	2.9±0.3	
	JHS	66 (20.89%)	2.8±0.4	
	SHS/vocational	98 (31.01%)	3.0±0.43	
	University/polytechnic	60 (18.99%)	3.6±3.6	
Occupation	Housewife	67 (21.20%)	3.5±0.9	0.001^b^
	Food seller	34 (10.76%)	3.0±0.3	
	Petty trading	77 (24.37%)	3.1±0.5	
	Seamstress/hairdresser	62 (19.62%)	2.9±0.3	
	Salaried worker	76 (24.05%)	3.0±0.4	
Parity	0	38 (12.03%)	2.8±0.5	0.001^b^
	1-3	233 (73.73%)	3.1±1.9	
	4-6	45 (14.24%)	2.9±0.2	
Ethnicity	Dagomba	146 (46.20%)	3.2±2.4	0.001^b^
	Gonja	56 (17.72%)	2.9±0.4	
	Mamprusi	42 (13.29%)	2.9±0.3	
	Ashanti/Ewe	26 (8.22%)	3.2±0.5	
	Others	46 (14.55%)	3.0±0.4	
Religion	Christian	66 (20.89%)	3.0±0.4	0.001^b^
	Muslim	238 (75.32%)	3.1±1.9	
	Traditionalist	12 (3.8%)	3.1±0.4	
Husbands' education	Primary	8 (2.53%)	2.9±04	0.002^b^
	JHS/middle school	18 (5.70%)	2.8±0.4	
	SHS	117(37.03%)	2.9±0.4	
	Tertiary	155(49.05%)	3.2±2.3	
	None	18 (5.70%)	3.0±0.51	
Husbands' occupation	Trader	50 (15.82%)	3.0±0.5	0.012^b^
	Employed	210 (66.46%)	3.1±1.9	
	Unemployed	7 (2.22%)	2.8±0.3	
	Farmer	37(11.71%)	2.9±0.35	
	Others	12 (3.80%)	2.7±0.5	
Weight gain/kg	Inadequate	56 (18%)	2.6±0.4	0.001^b^
	Adequate	176 (56%)	3.2±2.1	
	Excessive	83(26%)	3.1±0.3	

a : independent t -test; ^b^: one-way Anova; JHS: junior high school; SHS: senior high school

**Determinants of birth weight:** Table 6 illustrates the relationship between the dependent and independent variables. After controlling for confounders, maternal body mass index and weight gain during pregnancy were identified as predictors of low birth weight among pregnant women. The results showed that babies of mothers who were underweight before conception (BMI <18kg/m^2^) were more likely to deliver a low-birth-weight child (AOR=8.3, 95%CI: 6.7-15.0, p=0.001) compared to those who had normal weight. Furthermore, pregnant women who did not gain adequate weight during pregnancy were also more likely to give birth to a low birthweight child (AOR=4.5, 95% CI: 3.9-6.5, p<0.001) compared to those who gained adequate weight.

**Table 6:** determinants of birthweight

Variables	Category	^a^COR (95% CI)	P-value	^b^AOR (95% CI)	p-value
Age (years)	<20	8.05 (3.2-20.5)	0.001	5.3(0.35-81.4)	0.227
	20-30	Ref		Ref	
	30-40	0.5 (0.8-1.2)	0.134	0.9(0.15-5.7)	0.958
	>40	1		1	
Marital status	Married	Ref		Ref	
	Cohabiting	0.06(0.04-0.10)	0.001	16(0.09-276)	0.285
	Divorce/separated	1		1	
	Widowed	0.03(0.02-0.34)	0.005	0.01(0.002-9.1)	0.189
Parity	No child	7.7(3.5-17.4)	0.001	5.1(0.67-39)	0.115
	1-3	Ref		Ref	
	4-6	1		1	
Education	No education	1.5 (0.49-4.6)	0.477	0.94(0.04-17)	0.971
	Primary	1.5(0.47-5.0)	0.459	0.94(0.08-10)	0.960
	JHS	3.0(1.1-7.7)	0.020	3.9(0.39-40)	0.242
	SHS/vocational	Ref		Ref	
	University/polytechnic	1		1	
Husband occupation	Trader	0.8(0.5-1.3)	0.20	0.6(0.08-4.1)	0.589
	Employed	Ref		Ref	
	Unemployed	1.5(0.5-5.0)	0.56	22(1.7-29.01)	0.035
	Farmer	1.1(0.7-1.9)	0.43	1.27(0.08-19)	0.862
	Others	0.2(0.1-0.6)	0.63	4.5(0.04-408)	0.513
BMI (kg/m^2^)	Underweight	8.0(3.4-18.8)	0.001	8.3(6.7-15.0)	0.001
	Normal	Ref		Ref	
	Overweight	0.1(0.01-0.86)	0.035	0.12(0.01-1.9)	0.134
	Obese	0.45(0.06-3.6)	0.460	1.9(0.11-34)	0.640
Dietary diversity	Inadequate	8.3(3.3-20.0)	0.001	8.8(0.6-130)	0.11
	Adequate	Ref		Ref	
Socio-economic status	Low	10.1(4.3-23)	0.001	21(0.54-815)	0.102
	High	Ref		Ref	
Weight gain	Inadequate	4.2(1.4-13.1)	0.001	4.5(3.9-6.5)	<0.001
	Adequate	Ref		Ref	
	Excessive	1		1	

COR: crude odds ratio; AOR: adjusted odds ratio; ^a^: univariate regression; ^b^: multivariate regression; JHS: junior high school; BMI: body mass index; SHS: senior high school

## Discussion

The main aim of this study was to assess the effect of maternal dietary intake, gestational weight gain on birth weight. The study showed that mean dietary diversity score of pregnant women was 6.2 ± 0.1 SD, which is similar to finding by Ali *et al*. [45] who also reported a mean score (SD) of 6.17 (±0.99) and Acham *et al*. [46] reported a mean dietary diversity score (SD) of 6.70 (±2.22). It was established that the majority of participants in the current study had a diversified diet (more than 4 food groups), which is similar to Kenya based studies in which the majority of participants also had a diversified diet [45,47].

Furthermore, the consumption of meat and fish, as well as other fruits and vegetables, were high among study participants, contrary to other studies where the consumption of meat and meat products were low. For instance, pregnant women in Kenya were reported to consume low quantities of meat and meat products, meanwhile, in Bangladesh, 84.2% of pregnant women were reported to consume meat/fish products [48,49]. The high consumption of meat and fish among pregnant women in the Tamale Metropolis could be attributed to the location of irrigation dams near the metropolis and the rearing of livestock in and around the metropolis. Also, they take advantage of the rivers and irrigation dams to cultivate dry season gardens which provide them with sufficient vegetables like tomatoes and enough fruits. Ironically, with the availability of dry season gardens, vitamin A-rich vegetables, milk and milk products were among the least consumed among the study participants. This requires further investigation to unearth the reasons associated with low consumption of vitamin A-rich vegetables although vegetables are cultivated during dry season in the study area. These findings do not differ from Ochieng *et al*. [50] study in Tanzania where the majority of pregnant women did not consume milk and milk products.

In comparison with the national low birth weight estimated prevalence (13%), the estimated prevalence of low birth weight in the present study was lower. It is noteworthy to mention also that the prevalence ascertained in this study falls slightly outside of the WHO's target of less than 10% [51] but similar to the Multiple Indicator Cluster Surveys (MICS) survey prevalence (11%) in 2011. The prevalence obtained in this study could be as a result of improvements in health-related milestones in the northern region as reported in the Ghana demographic and health survey reports [52]. Macrosomia births reported in the present study is lower than reported in other studies conducted in Ghana [18,25].

In identifying predictors of low birth weight among pregnant women, maternal pre-pregnancy BMI and weight gain during pregnancy were identified as predictors of low birth weight. The study realized that maternal pre-pregnancy BMI to a large extent determined the birth weight of babies delivered by respondents. Pregnant mothers who were underweight before pregnancy were at higher risk of delivering babies with low weight compared to those who were having a normal body mass index. The above finding was in line with literature which showed that there exists an association between maternal pre-pregnancy BMI and the birth weight of new-borns [53,54]. Similarly, a study found in Beijing, China, reported that pregnant women with inadequate weight before pregnancy were at higher risk of having a low birthweight baby [55]. Available literature attributes the association between low birth weight and a BMI of less than 18.5kg/m^2^to negative energy balance. In the same vein, LBW was higher in infants whose mothers were underweight during pregnancy compared to those whose mothers were normal. A systematic analysis of 42 studies showed that offspring born to underweight mothers were at greater risk of having LBW compared to those born to women with an average weight in both developed and Low Middle-Income Countries (LMICs) [56]. This finding is additionally supported by a recent meta-analysis within the context of LMICs, revealed that LBW was significantly associated with maternal underweight, but not maternal overweight/obesity [57]. These findings, therefore, require strong policy attention to address the problem of undernutrition among mothers, especially for the rationale of its intergenerational effects. This in effect presents adverse consequences on birth weight and infant and maternal mortality [58]. As undernutrition in itself a multifactorial problem, the solution would require developing cross-cutting policies and placing the issue on a broad national health and development agenda.

Lastly, the study also found an association between pregnancy weight gain and the birth weight of the child. Mothers who gained inadequate weight were more likely to deliver low-weight babies compared to their counterparts who gained adequate weight. This finding is consistent with other studies conducted in many parts of the world. For instance, a study conducted by Lima *et al*. [59] in Brazil found that the effect of gestational weight gain on the increase in birth weight was greater than that of pre-pregnancy body mass index (P-value of 0.001). Similar findings have been reported by studies conducted in Beijing, China, that women with inadequate gestational weight gain had a higher risk of low birth weight and small for gestational age infants [55]. Moreover, several studies have shown that the birth weight of the baby was significantly associated with gestational weight gain [60,61].

As with cross sectional studies, the cause-effect relationship is not intended. Besides cross-sectional studies have inherent recall bias. However, this was unlikely to alter the results of the present study as effort was made assist respondents to provide appropriate responses through probing. Also, the study was conducted among women who attended ANC and delivered in a health facility. Since some women still give birth in their homes in Northern Ghana, the generalisation of the findings emanating from this study should be done with caution. As with retrospective study, the study was prone to recall bias. To mitigate this, the study participants selected mothers who were attending postnatal care services at selected health facilities in the metropolis. With this most of the data were recorded already in the maternal and child health record book. Moreover, effort was made to assist respondents to provide appropriate responses through probing, hence recall bias was unlikely to alter the results of the present study.

## Conclusion

The study showed that a double burden of malnutrition (low birth weight and macrosomia) coexisted among new-borns in the Tamale Metropolis, which is currently being experienced by developing and transition counties. Pre-pregnancy BMI and weight gain during pregnancy were found to predict birth weight. Low birth weight is a major public health concern and the causes are multifaced in natures. Therefore, in order to deal with it a more holistic and multi-sectoral approaches such as behaviour change communication and comprehensive preconception care are required.

### What is known about this topic

- Dietary consumption during pregnancy has a significant impact on the growth and development of the fetus;
- Pregnancy-related physiological changes make it difficult to maintain a healthy diet, which has serious detrimental effects on both the mother and the growing fetus;
- Adverse maternal and fetal effects such as large gestational age (LGA) babies, gestational diabetes mellitus (GDM), caesarean delivery, early pregnancy loss, preeclampsia, and postpartum weight retention result from excessive weight gain.

### What this study adds

- The double burden of malnutrition coexisted among infants born in the Tamale Metropolis comprising of low birth weight (undernutrition) and macrosomia (overnutrition), which is currently being experienced by developing and transition counties;
- This research also reveals valuable details on the birth weight determinants of babies born in the Tamale Metropolis;
- Maternal body mass index and weight gain during pregnancy were strong predictors of low birth weight among pregnant women.
